# One Single Session to Sleep Them All? The Potential of Single‐Session Interventions for Insomnia

**DOI:** 10.1111/jsr.70134

**Published:** 2025-06-30

**Authors:** Matteo Carpi, Erica Marie Szkody, Daniel Ruivo Marques

**Affiliations:** ^1^ Department of Human Neuroscience Sapienza University of Rome Rome Italy; ^2^ Department of Medical Social Sciences, Feinberg School of Medicine Northwestern University Chicago Illinois USA; ^3^ Department of Education and Psychology University of Aveiro Aveiro Portugal; ^4^ CINEICC—Centre for Research in Neuropsychology and Cognitive Behavioral Intervention, Faculty of Psychology and Educational Sciences University of Coimbra Coimbra Portugal

**Keywords:** cognitive behavioural therapy for insomnia (CBT‐I), digital interventions, insomnia, mental health, single‐session interventions

## Abstract

Poor sleep and insomnia are pervasive public health concerns, with insomnia ranking as the second most prevalent mental health disorder in the general population. Moreover, insomnia is a significant predictor of subsequent depression and is frequently co‐occurring with other psychological difficulties. While both in‐person and digital cognitive‐behavioural treatments have proven effective as first‐line strategies for managing insomnia, their accessibility remains limited, with several barriers preventing dissemination, particularly, among at‐risk or hard‐to‐reach populations such as adolescents, university students and minority groups. Single‐session interventions (SSIs) for mental health have recently emerged as a strategic asset and treatment approach, grounded in specific theoretical tenets and assumptions. The efficacy and feasibility of SSIs have been well documented, with systematic evidence highlighting their flexibility, applicability and cost‐effectiveness across a wide range of clinical and subclinical conditions. However, their full implementation in the treatment of insomnia remains limited. This brief review aims to illustrate the alignment between the SSI framework and the needs of psychological treatments for insomnia and to summarise current evidence on single‐session, one‐shot interventions for insomnia. Notably, several one‐shot interventions based on cognitive‐behavioural therapy for insomnia have been tested in clinical trials with promising results, though their integration with the broader SSI approach appears partial. Further research is warranted to develop consensus‐based in‐person and digital SSIs for insomnia and to assess their feasibility and effectiveness within a stepped‐care framework.

## 1 There and Back Again: Psychological Treatment of Insomnia

With over 10% of the adult population meeting the diagnostic criteria for chronic insomnia disorder worldwide (Aernout et al. 2021; Morin et al. 2015; Morin and Jarrin 2022; van Straten et al. 2025), it is currently recognised as the second most prevalent mental health condition (Mao et al. 2024). Given insomnia's broad impact on both mental and physical health (Benz et al. 2023; Fernandez‐Mendoza and Vgontzas 2013), awareness has grown regarding the advantages of early treatment and prevention (Hale et al. 2020; Hertenstein et al. 2016). Indeed, given the tight and bidirectional relationship between insomnia and the depression–anxiety spectrum (Hertenstein et al. 2023; Jansson‐Fröjmark and Lindblom 2008), addressing clinical and subclinical sleep complaints may serve as a strategic intervention to prevent the onset of further psychopathology in the long term (Palagini et al. 2024). Against this backdrop, psychological and behavioural treatments have historically demonstrated their feasibility and effectiveness in treating insomnia, with cognitive behavioural therapy for insomnia (CBT‐I) now representing the first‐line, gold‐standard approach, yielding superior long‐term outcomes compared to pharmacological treatments (Edinger et al. 2021; Qaseem et al. 2016; Riemann et al. 2023).

In fact, robust clinical research has repeatedly reinforced the merits of psychological interventions (Alimoradi et al. 2022; Trauer et al. 2015; Zachariae et al. 2016; Zhang et al. 2022) in a field where traditional medications (i.e., benzodiazepines and benzodiazepine receptor agonists known as Z‐drugs, along with more recent dual orexin receptor antagonists) often fail to achieve sustained clinical effectiveness and are not considered suitable for chronic use (Riemann et al. 2023; Sateia et al. 2017). Moreover, the models underpinning CBT‐I have been developed in alignment with the clinical conceptualisation of the processes leading to the onset and maintenance of insomnia disorder—namely, predisposing and perpetuating factors (i.e., Spielman's 3Ps framework; Spielman et al. 1987; Wright et al. 2019)—and can be readily situated within models that account for pathophysiological evidence on the mechanisms of insomnia, including physiological, cognitive and emotional hyperarousal (Dressle and Riemann 2023).

Notably, CBT‐I can be delivered in individual, group or digitally assisted formats (Gao et al. 2022) and is structured in a modular fashion (i.e., composed of separable behavioural and cognitive components such as stimulus control, sleep restriction and cognitive therapy; Edinger and Carney 2015). This structure allows for tailored interventions adapted to different clinical phenotypes and severity levels within a stepped‐care framework (Baglioni et al. 2023; Vincent and Walsh 2013). However, its dissemination remains challenging, considering the large proportion of individuals for whom treatment would be advisable (Manber et al. 2015; Muench et al. 2022). Barriers such as a lack of trained practitioners, limited resources and adherence and attrition issues (Crawford et al. 2016; Simon et al. 2025) have driven the development of abbreviated protocols (e.g., two‐session abbreviated therapy for insomnia or four‐session brief behavioural therapy for insomnia; Edinger and Sampson 2003; Troxel et al. 2012) as well as self‐help and digitally delivered adaptations of traditional CBT‐I, both of which have demonstrated effectiveness in controlled studies (Ho et al. 2015; Kwon et al. 2022).

Yet, CBT‐I and other evidence‐based protocols remain largely inaccessible, and even when available, non‐response to treatment is not uncommon, with therapeutic response observed in approximately 70%–80% of patients and remission rates at follow‐up ranging from 40% to 60% (Morin and Benca 2012; Muench et al. 2022). This reality has pushed the field to reflect on opportunities to optimise psychological treatments for insomnia further, making them as accessible and feasible as possible—in part by engaging in dialogue with implementation science (Angelillo et al. 2025). Accordingly, a growing research trend has emerged, aiming to mechanistically explore which single treatment components are most effective in reducing insomnia symptoms and how they can be scaled for dissemination in public health contexts (Kyle et al. 2015; Maurer et al. 2018; Steinmetz et al. 2024). This direction is exemplified by a recent large clinical trial demonstrating the feasibility and efficacy of single‐component sleep restriction therapy delivered by trained nurses (Kyle et al. 2023).

As will be shown in this perspective, this line of investigation—aimed at maximising the cost‐effectiveness of insomnia treatments by wisely applying Occam's razor—is well aligned with the growing movement of single‐session interventions (SSIs) in mental health (Schleider et al. 2020; Schleider and Weisz 2017). Indeed, as will be further discussed, SSIs may be particularly suitable as early interventions, for subclinical presentations or within stepped‐care models where accessibility and rapid delivery are crucial. Several studies have already demonstrated the efficacy of very brief or one‐shot adaptations of traditional CBT‐I, even in comparison with longer protocols (Edinger et al. 2007; Ellis et al. 2015; Kwon et al. 2022). Notably, SSIs can be offered at various stages of care (e.g., as standalone interventions or as supportive modules aiding access, bridging ongoing care or assisting treatment termination) and may also prove more accessible to individuals seeking mental health support online(Schleider et al. 2025), a population that often remains underserved in sleep‐focused care pathways.

In exploring the feasibility and potential of implementing SSIs for the prevention and treatment of insomnia, this narrative review will illustrate the core principles of the SSI approach and provide a state‐of‐the‐art synthesis of previous proposals and experiences with one‐shot psychological interventions for insomnia. This material will be framed as a starting point for future implementation of SSIs in the field of behavioural sleep medicine.

Specifically, the article is structured into three core sections. First, we outline the theoretical foundations of SSIs in psychotherapy and their application within mental health. Next, we present a narrative synthesis of existing SSIs for insomnia, highlighting the key features and outcomes of available one‐session protocols. Finally, we discuss the potentials and limitations of SSIs in insomnia treatment at the intersection of sleep medicine and psychotherapy, with a focus on implications for future research and clinical practice.

## 2 Riddles in the Dark: The SSIs Wave in Mental Health

While single‐session or (often forcedly) abbreviated therapies have existed since the early days of psychotherapy (Dryden 2023), deliberate efforts to provide effective therapeutic interventions within a single encounter have gained traction more recently (Schleider et al. 2025). This shift followed research‐backed recognition that many psychological treatments designed to span multiple sessions often conclude after just a handful of meetings—or even a single session (Abel et al. 2022; Olfson et al. 2009; Talmon 1990; Wells et al. 2013). In line with this trend, systematic evidence has been gathered and SSIs have emerged as a standalone therapeutic approach with distinct conceptual foundations and assumptions (Schleider et al. 2020; Talmon 2012). Indeed, the efficacy and feasibility of SSIs for mental health issues, particularly anxiety and depression, have been shown to be comparable to those of longer, traditional psychotherapies. Systematic reviews and meta‐analyses demonstrate their flexibility, applicability and cost‐effectiveness across a wide range of clinical and subclinical conditions, with, particularly, striking success in youth populations (Schleider et al. 2025; Schleider and Weisz 2017). With a Hedges' *g* effect size of 0.32, based on randomised clinical trials involving a pooled sample of 10,508 youths with various psychiatric problems, SSIs demonstrated better outcomes than control conditions with a 58% probability (Schleider and Weisz 2017). While a detailed overview of condition‐specific evidence is beyond the scope of this perspective, it is worth summarising the cornerstone principles that unify these interventions and defining SSIs as an ‘approach’ to mental health treatment.

SSIs are currently defined as structured programmes that are specific in their aims and intentionally designed to deliver a single visit, encounter or interaction with a clinic, provider or digital platform, embracing various formats and modalities (e.g., in‐person, digitally delivered, in‐app) (Schleider et al. 2020). While the intentionality of pursuing a concentrated format is essential, the theoretical orientation is not fixed, nor are the target clients or settings. SSIs employ diverse techniques –cognitive‐behavioural, person‐centred or solution‐focused–delivered to individuals, families or groups in hospitals, clinics, schools or at home. At their core, these interventions typically leverage the same mechanisms as longer treatments (e.g., behavioural activation or cognitive restructuring for depression, communication skills training to foster healthy romantic relationships; Schleider et al. 2021, 2023; Steinberg et al. 2024) packaged in a skill‐training format with purposeful action planning. Moreover, SSIs are accompanied by motivation‐enhancing, transtheoretical strategies that include psychoeducation and normalisation of the client's experience, empowerment of clients as ‘experts’ in managing their issues, encouragement to share newly acquired skills within their communities, and the use of narratives from others who have faced similar challenges (Schleider and Beidas 2022).

This core structure places strong emphasis on treatment acceptability and allows for straightforward implementation of highly scalable SSIs across different targets and delivery formats (both in‐person and fully digital, unassisted) (Cohen et al. 2024; Schleider and Beidas 2022), weaving together scientific evidence on both specific psychotherapeutic techniques (or therapeutics, as recently proposed in CBT‐I literature; Espie 2023) and psychotherapy common factors, including relational, community and client‐related elements (Ahuvia et al. 2023; Laska et al. 2014). Arguably, these factors, as well as systemic considerations of treatment enhancement and broader contextual influences, have been only marginally integrated into traditional cognitive behavioural treatments in sleep medicine. Their introduction could represent a major contribution from aligning behavioural sleep medicine more closely with the SSIs movement.

## 3 Journey to the Cross‐Roads: One‐Shot Interventions for Insomnia

As already discussed, the abbreviation of treatment protocols has been proposed as a response to the barriers preventing the widespread dissemination of CBT‐I. However, well‐established variations of traditional CBT‐I like brief behavioural treatment of insomnia and the majority of digital CBT‐I programmes still typically span several structured sessions. To summarise current experiences with single‐session, one‐shot interventions for insomnia, we conducted a literature search on MEDLINE/PubMed from inception to March 2025, with the aim of identifying studies readily accessible to clinicians and practitioners (i.e., original articles and reports published in peer‐reviewed journals by the time of the search). The comprehensive query string used was: [(‘brief‘ OR ‘abbreviated‘ OR ‘one‐shot‘ OR ‘single‐session‘) AND ((‘CBT‐I‘ OR ‘cognitive behavioural therapy for insomnia‘ OR ‘cognitive behavioural therapy for insomnia‘ OR ‘behavioural therapy for insomnia‘ OR ‘behavioural therapy for insomnia‘ OR ‘insomnia treatment‘ OR ‘insomnia intervention‘ OR ‘brief behavioural treatment for insomnia‘ OR ‘digital CBT‐I‘ OR ‘dCBT‐I‘)]. Reference lists of screened records were also checked to identify additional relevant publications, and corresponding authors were contacted when information was missing or unclear. Before conducting this preliminary scoping review, targeted searches of the PROSPERO registry (https://www.crd.york.ac.uk/PROSPERO/) and the Open Science Framework (OSF; https://www.osf.io) did not identify any existing or ongoing reviews on this topic.

In line with the exploratory aims of this narrative synthesis, we considered all types of research records (e.g., clinical trials, study protocols and qualitative investigations) reporting on SSIs for insomnia and/or general sleep complaints. No formal quality assessment of the included studies was performed. A schematic summary of the nine retrieved studies is presented in Table 1, while a narrative overview is provided within this section. Given the limited scope and marked heterogeneity of the available evidence, no quantitative synthesis was attempted.

**TABLE 1:** Summary of reviewed studies investigating single session (one‐shot) interventions for insomnia.

Authors (year)	Country	Study design	Sample	Mean age	Sex (% women)	Intervention	Treatment components	Main findings
Amra et al. (2023)	Iran	RCT	57 healthcare workers with acute insomnia (*n* = 31 intervention vs. *n* = 26 inactive control)	34.6 (intervention) vs. 36.6 (control)	81% vs. 65%	Small group sessions (2–3 participants)	Stimulus control, restructuring maladaptive behaviours/cognitions about sleep, sleep restriction	Reduction in insomnia severity and increased high‐frequency HRV
Boullin et al. (2016)	United Kingdom	Two‐arm pilot study (self‐assignment to individual or group treatment)	25 participants with acute insomnia (*n* = 13 individual format, *n* = 12 group format)	42.0 (individual) vs. 39.6 (group)	76.9% vs. 75%	One‐shot CBT‐I in individual or group setting + self‐help pamphlet (format adapted from Ellis et al. 2015)	Sleep education, sleep hygiene, restructuring dysfunctional beliefs about sleep, sleep restriction, stimulus control, cognitive control, imagery distraction	Comparable positive effects between individual and group delivery
Chevalier et al. (2023)	United States of America	Single‐arm study	34 cancer survivors	N.A.	79%^a^	75‐min STEP‐1 workshop (in‐person or videoconference)	Sleep hygiene, stimulus control, cognitive restructuring; focus on stimulus control, bed timing and action plan	Reduced insomnia severity; high acceptability; no differences between in‐person or videoconference format
Crosby et al. (2025)	United States of America	RCT	61 university students (*n* = 31 intervention vs. *n* = 30 active control)	19.9 (intervention) vs. 19.6 (active control)	71% vs. 90%	Self‐guided, internet‐based programme (sleep scholar)	Sleep education, stimulus control, sleep restriction; interactive content	High satisfaction; no superior efficacy of Sleep Scholar over active control; improvements in both conditions
Edinger et al. (2007)	United States of America	RCT (dose–response trial of different numbers of CBT‐I sessions)	86 (*n* = 16 one session CBT‐I, *n* = 18 two sessions, *n* = 24 four sessions, *n* = 17 eight sessions vs. *n* = 11 waitlist)	55.9 (one‐session), 55.5 (two‐session), 57.0 (four‐session), 54.7 (eight‐session), 52.4 (waitlist)	50%, 55.6%, 50%, 52.9%, 36.4%	In‐person individual CBT‐I, single session	Sleep education, stimulus control, sleep restriction	One‐ and four‐session formats most effective, outperforming 2‐ and 8‐session formats and waitlist
Ellis et al. (2015)	United Kingdom	RCT	40 adults with acute insomnia (*n* = 20 single session vs. *n* = 20 waitlist)	32.9 (single session) vs. 31.8 (waitlist)	55% (in both groups)	Single face‐to‐face session CBT‐I + self‐help pamphlet	Sleep education, sleep hygiene, restructuring dysfunctional beliefs about sleep, sleep restriction, stimulus control, cognitive control, imagery distraction	Significant reduction in insomnia severity and higher clinical remission in the intervention group at 1‐month follow‐up
Maity et al. (2024)	United Kingdom	Qualitative study (user experience)	11 adolescents	18.2	72.7%	Prototype online self‐help SSI for adolescent sleep problems	Sleep education, sleep hygiene, stimulus control, arousal reduction, normalisation, empowerment, action planning	Positive feedback on the SSI; emerging themes for SSI user experience: balance (‘educative‘), usability (‘effortless‘), personalisation and positive outlook (‘positivity‘)
Okun and Glidewell (2023)	United States of America	Single‐arm study	45 adults seeking treatment for self‐reported insomnia	53.8	71.1%	4‐h group CBT‐I workshop by health educator	Sleep education, sleep diaries, stimulus control, sleep restriction, cognitive restructuring, relaxation, mindfulness	Improved insomnia symptoms; reduced use of sleep aids; 20% clinical remission
Randall et al. (2019)	United Kingdom	Single‐arm open trial	30 male offenders	33.1	0% (all male)	Individual one‐shot CBT‐I in prison setting + self‐help pamphlet (format adapted from Ellis et al. 2015)	Sleep education, sleep hygiene, restructuring dysfunctional beliefs about sleep, sleep restriction, stimulus control, cognitive control, imagery distraction	Significant reductions in insomnia severity, depression and anxiety symptoms

Abbreviations: CBT‐I cognitive behavioural therapy for insomnia; N.A.: not available; RCT: randomised controlled trial; SSI: single session intervention.

^a^ This information was not reported in the text and was obtained directly from the corresponding author of the paper.

In a seminal study investigating the dose–response effects of in‐person, individual CBT‐I, Edinger et al. (2007) conducted a randomised controlled trial comparing different treatment lengths (one single session, two, four and eight sessions) to a waiting list control in 86 adults with sleep‐maintenance insomnia. The single‐session format included education on sleep needs, circadian rhythms and dysfunctional sleep beliefs, along with instructions for correcting sleep‐disrupting habits, stimulus control and simplified sleep restriction prescriptions based on sleep diaries. In the other treatment formats, subsequent sessions provided additional guidance on sleep restriction titration and encouragement for adherence. Notably, the one‐ and four‐session formats were the most effective, both subjectively and objectively, outperforming the waiting list, two‐session CBT‐I and even the eight‐session CBT‐I.

Focusing on acute insomnia (i.e., insomnia symptoms lasting less than 3 months), Ellis et al. (2015) developed an ad‐hoc condensed version of CBT‐I consisting of a single face‐to‐face session, augmented by a self‐help pamphlet, tested in a randomised controlled trial versus a waiting list in 40 adults. During this one‐shot encounter, participants received education about sleep and insomnia and sleep hygiene practices and dysfunctional beliefs were discussed. The session's core focus was the introduction and explanation of sleep restriction, with individualised prescriptions based on pre‐treatment sleep diaries. At the end of the session, participants were provided with a pamphlet containing practical instructions on stimulus control, cognitive control and imagery distraction techniques for coping with episodes of insomnia. At one‐month follow‐up, the intervention group showed significantly reduced insomnia severity and higher clinical remission rates compared to the control group.

Promisingly, the same protocol was investigated in two subsequent pilot studies. In the first, comparable effects were found in 25 adults with acute insomnia who received one‐shot CBT‐I either in individual or group format (Boullin et al. 2016). In the second, the protocol was delivered individually to 30 male offenders during their stay in prison, resulting in significant, large effect‐size reductions in insomnia severity, as well as improvements in depressive and anxiety symptoms (Randall et al. 2019).

A similar one‐shot CBT‐I format was investigated in a randomised controlled trial versus an inactive control group involving 57 healthcare workers with acute insomnia during the COVID‐19 pandemic (Amra et al. 2023). Participants were required to complete sleep diaries in the 2 weeks preceding the intervention, which was delivered to small groups of two or three of them. The session provided information on stimulus control techniques, maladaptive behaviours and cognitions contributing to the development of chronic insomnia and instructions for applying sleep restriction. After treatment, the group receiving the intervention demonstrated a more pronounced reduction in self‐reported insomnia severity, along with an increase in high‐frequency heart‐rate variability—potentially reflecting enhanced parasympathetic nervous system activity and a concomitant reduction in sympathetic hyperactivity.

In another single‐arm study, the effects of a single‐session CBT‐I group workshop delivered by a health educator were tested in 45 adults with self‐reported insomnia (Okun and Glidewell 2023). The 4‐h workshop encompassed all the core components of CBT‐I: sleep education, sleep diary completion, stimulus control, sleep restriction (calculated based on mean self‐reported sleep duration rather than pre‐treatment diaries), cognitive interventions and stress reduction techniques, including relaxation and mindfulness exercises. At 1‐month follow‐up, significant improvements in self‐reported insomnia symptoms were observed, alongside a reduction in the use of sleep aids. Most participants reported better sleep quality and 20% achieved clinical remission.

The effectiveness and feasibility of a similar protocol—the Sleep Treatment Education Program‐1 (STEP‐1), an educational workshop delivered either in person or via videoconference—were specifically evaluated in 34 cancer survivors, given the high prevalence of insomnia in this population (Chevalier et al. 2023). The 75‐min, instructor‐led intervention emphasised behavioural change by presenting key components of CBT‐I, though it deliberately excluded sleep restriction to minimise patient burden, instead focusing on stimulus control and bed timing. Participants were encouraged to create an individualised sleep action plan to select information most relevant to their personal goals and anticipate potential barriers. At one‐month follow‐up, insomnia severity significantly decreased, with no differences between delivery formats. Moreover, the majority of participants reported improved sleep quality and all those who responded to programme‐evaluation questions stated they would recommend the workshop to others facing their situation and challenges.

Another single‐session sleep intervention, Sleep Scholar, was specifically designed for university students and tested in a randomised controlled trial versus an active control intervention promoting general healthy habits enrolling 61 students reporting at least subclinical insomnia symptoms (Crosby et al. 2025). The intervention was self‐guided, Internet‐based and did not involve a therapist or educator, consisting of three brief modules focusing on sleep education, stimulus control and sleep restriction. The modules were delivered as a single online session within a browser‐based environment, with a mean completion time of approximately 33 min reported in a preceding feasibility pilot study (Crosby and Witte 2023). Interactive material included text, animations, vignettes and quizzes. At post‐treatment, the group receiving Sleep Scholar reported higher satisfaction and rated the programme as a more acceptable treatment for insomnia compared to the control intervention. However, at one‐week and one‐month follow‐ups, Sleep Scholar did not demonstrate superior efficacy in improving insomnia, subjective sleep quality or mental health symptoms (depression, anxiety, posttraumatic stress and suicidal ideation) compared to the control condition, with both groups showing significant improvements in sleep outcomes and depression, anxiety and posttraumatic stress symptoms.

Finally, a recent study explored user experience with a prototype online SSI for sleep problems in adolescents (Maity et al. 2024). Notably, the intervention was developed following the SSI principles previously described (Schleider and Beidas 2022), integrating core components of cognitive and behavioural protocols for insomnia (i.e., sleep education, sleep hygiene, stimulus control and arousal reduction, but not sleep restriction) into a framework that emphasised normalisation, psychoeducation, empowerment (through peer‐to‐peer behavioural advice generation) and action planning (defining future steps and anticipating challenges). Although efficacy data were not collected, participants' opinions on the quality of the intervention were obtained and user experience information was gathered via think‐aloud interviews with 11 adolescents who completed the programme. Thematic analysis of the interviews highlighted the intervention's perceived helpfulness, with key themes including the balance between educational content and enjoyable activities, ease of use and time efficiency, a positive outlook and opportunities for personalisation. Although preliminary, this in‐depth exploration of user experience represents one of the few existing examples of intensive participant feedback integration into the conceptualisation of a one‐shot insomnia intervention—a process that could foster a virtuous cycle of refinement and improvement in future implementation efforts.

Taken together, the reviewed studies suggest that SSIs for insomnia are feasible across a range of settings—including clinical, institutional and educational contexts—and delivery modalities. Promising effects have been reported in acute, subclinical and comorbid insomnia presentations. Intervention components commonly included sleep education, stimulus control and sleep restriction, although the extent to which cognitive and behavioural elements were integrated varied substantially. Overall, the available evidence supports SSIs as a potentially effective and scalable approach for early intervention or as an adjunct to other treatments, though findings remain heterogeneous and preliminary.

## 4 Conclusions and the Next Journey

At the end of this exploration, the time seems ripe for insomnia research to seriously invest in SSIs. While the framework for developing and testing SSIs in various formats is now well established, the literature on one‐shot interventions for insomnia is still emerging. Overall, current evidence is promising in demonstrating the clinical efficacy of very brief programmes, whether delivered in‐person or online, in both one‐to‐one and group settings. However, further investigation appears warranted for self‐guided digital solutions, which arguably represent the most scalable and cost‐effective form of SSIs (Jans et al. 2024).

As insomnia continues to place a substantial burden on individuals and healthcare systems (Morin and Jarrin 2022; van Straten et al. 2025)—and access to evidence‐based care remains constrained—there is a timely opportunity to expand highly scalable, low‐intensity interventions such as SSIs. This is particularly relevant within stepped‐care frameworks and task‐shifting strategies (Baglioni et al. 2023; Espie 2023; Schleider and Beidas 2022), where early, efficient therapeutic encounters can improve reach and equity of care delivery.

In terms of treatment content, all interventions to date have drawn on multiple components of CBT‐I, with most focusing primarily on the implementation of sleep restriction. Yet, given the positive outcomes reported by Chevalier et al. (2023) in a protocol that did not include sleep restriction, the differential impact of SSIs for insomnia with or without sleep restriction is certainly worth investigating. This consideration is, particularly, relevant in light of potential difficulties with adherence, dropouts linked to sleep timing prescriptions and the challenge of obtaining pre‐treatment sleep diaries. Moreover, sleep restriction without guidance may be difficult to implement and could be contraindicated or require close monitoring in specific populations, such as shift workers or patients with a history of manic episodes (Järnefelt and Spiegelhalder 2022; Miller et al. 2014). Nevertheless, sleep restriction therapy and stimulus control arguably remain the most robust, evidence‐based and straightforward components of CBT‐I (Furukawa et al. 2024; Miller et al. 2014) and their inclusion in SSI protocols is strongly supported. Future research may benefit from testing different combinations of components—whether multi‐component or single‐technique—tailored to the delivery format, guidance level and target population.

When examined through the lens of the guiding principles of the SSIs approach (Schleider et al. 2020), current one‐at‐a‐time interventions for insomnia appear successful in implementing psychoeducation, fostering skills‐building (i.e., teaching and practicing behavioural and cognitive techniques) and promoting action planning (e.g., framing newly acquired skills within future strategies to manage setbacks such as bad nights or sleep‐related worries). Furthermore, although SSIs can be delivered in either digital or in‐person formats, they are conceptually distinct from digital multi‐session CBT‐I programmes. While the latter involve structured engagement over time—sometimes with therapist support—SSIs aim to deliver a concentrated therapeutic dose in a single encounter. This distinction is important when evaluating cost‐effectiveness, therapist time and adherence, areas where SSIs may hold particular advantages for initial engagement or outreach (Schleider and Beidas 2022). Moreover, meta‐analytic data show comparable effect sizes for human‐delivered and fully self‐guided (digital or paper‐based) SSIs in youth (Schleider and Weisz 2017) and recent evidence suggests that shortening the duration of digital SSIs does not diminish effectiveness (Kaveladze et al. 2025), underscoring the adaptability of well‐designed, ultra‐brief treatment formats. Nonetheless, empirical comparisons between digital SSIs and multi‐session digital interventions remain limited and warrant further investigation to clarify their respective strengths and shortcomings. On the other hand, strength‐based strategies, user empowerment and the sharing of experiences with peers or through testimonials and community narratives are less frequently incorporated, despite evidence supporting their potential to enhance both effectiveness and acceptability (Schleider and Beidas 2022). A dedicated design approach capitalising on user feedback and drawing from prior successful SSI experiences could help address these gaps. For example, the growth mindset concept based on brain plasticity, successfully employed in SSIs for youth mental health (Schleider and Weisz 2018), could be easily adapted to insomnia interventions to illustrate the transformative effects of behavioural change on sleep regulation. Similarly, incorporating value‐based action and life‐values assessment, as proposed in the Project ABC SSI (Schleider et al. 2021), could boost motivation to change sleep‐related behaviours. Remarkably, this focus aligns well with recent tendencies towards integrating third‐wave cognitive behavioural principles, such as those found in acceptance and commitment therapy, into insomnia treatments (Angelillo et al. 2025).

Taken together, these considerations call for insomnia clinical research to align with the broader trend of improving the scalability and accessibility of psychological interventions. With data from the nationwide UK National Health Service Improving Access to Psychological Therapies initiative showing that up to 47% of patients either do not attend assessment or discontinue treatment after the first session (thus never accessing even low‐intensity, two‐session interventions) (Richards and Borglin 2011), SSIs are well positioned to serve as a potential first gate to mental health care.

Translating this into the stepped‐care framework proposed for insomnia treatment (Baglioni et al. 2023), SSIs could represent the first step for individuals presenting with insomnia‐related complaints, either as a standalone intervention or as an initial therapeutic encounter preceding further psychological or pharmacological treatment.

While it is unlikely that SSIs, in their current form, will match the long‐term effectiveness of full multi‐session CBT‐I—particularly, for individuals with chronic or treatment‐resistant insomnia—the existing results are nonetheless encouraging. As a supplementary exploration, we examined three studies from this review that featured a between‐group, multiple‐arm design comparing SSIs to control conditions and that reported sufficient data for effect size estimation—namely, Amra et al. (2023), Edinger et al. (2007) and Ellis et al. (2015) (see Supporting Informations for details). Effect sizes in these studies were consistent with benchmark estimates reported in meta‐analyses of full or abbreviated CBT‐I (Kwon et al. 2022; van der Zweerde et al. 2019). Notably, in Edinger et al. (2007), the single‐session CBT‐I format demonstrated a comparable effect size to the full eight‐session protocol, was superior to the two‐session condition and was only outperformed by the four‐session protocol. While these findings are based on limited sample sizes and cannot be generalised beyond their specific contexts, they point to the potential of SSIs as a viable, efficient alternative in stepped‐care models. Further research is needed to confirm their comparative effectiveness across diverse clinical populations and implementation settings.

Therefore, this ‘heavy luggage‘ of potential and promise must now be carried into the next journey. With guidelines already in place (Manvelian et al. 2025; Schleider and Beidas 2022), the field now faces the task of developing and evaluating consensus‐based SSIs for insomnia, with an emphasis on enabling quantitative synthesis of their outcomes. Particular emphasis should be placed on designing well‐grounded digital SSIs aimed at serving at‐risk and hard‐to‐reach populations, such as youth, minority groups and individuals facing traumatic experiences, for whom the prevention or mitigation of insomnia may represent a relevant strategic goal (Hertenstein et al. 2016; Perlis et al. 2021). Ultimately, exploring the feasibility and effectiveness of (alternative protocols of) SSIs across diverse insomnia phenotypes and comorbidity profiles (Nyhuis and Fernandez‐Mendoza 2023) stands out as an ambitious long‐term objective.

By facing these challenges with curiosity and a growth mindset, sleep researchers and clinicians may soon unveil the full potential of SSIs for insomnia.

## Author Contributions

**Matteo Carpi:** conceptualization, investigation, data curation, formal analysis, methodology, visualization, writing – original draft, writing – review and editing. **Erica Marie Szkody:** writing – review and editing, visualization, supervision. **Daniel Ruivo Marques:** visualization, supervision, writing – review and editing.

## Conflicts of Interest

The authors declare no conflicts of interest.

## Supporting information


**Data S1.**Supporting Information.

## Data Availability

Data sharing is not applicable to this article as no new data were created or analyzed in this study.
