# Supplementary material for: One Single Session to Sleep Them All? The Potential of Single‐Session Interventions for Insomnia
Source: J Sleep Res. 2025 Jun 30;35(2):e70134. doi: 10.1111/jsr.70134 (PMC13003307; doi:10.1111/jsr.70134)
Supplement: Supplementary file 1 — Data S1.Supporting Information. [file JSR-35-e70134-s001.docx]

*Supplementary Materials*

**One single session to sleep them all? The potential of single-session interventions for insomnia**

Matteo Carpi, Erica Marie Szkody, and Daniel Ruivo Marques

**Supplementary analysis: effect sizes from multiple-arm controlled studies on single-session interventions for insomnia**

*Multiple-arm controlled studies included in the review*

This supplementary section presents a focused analysis of between-group effect sizes (i.e., treatment vs. control) derived from studies included in the review that featured a multiple-arm trial with at least one single-session cognitive-behavioural therapy for insomnia (CBT-I) intervention. Only studies that reported sufficient data for effect size computation (i.e., baseline and post-treatment means and standard deviations) were included. was used as the reference outcome measure due to its wide availability and frequent inclusion across the reviewed studies (see **Table 1** in the main text for an overview of included studies).

After careful examination, three studies employing a multiple-arm design were identified as eligible for effect size computation, namely:

- Amra et al. (2023): A randomised controlled trial evaluating the efficacy of a one-shot CBT-I intervention versus a no-intervention control group in participants with self-reported insomnia symptoms.
- Edinger et al. (2007): A randomised controlled trial comparing different "doses" of CBT-I – including one, two, four, and eight sessions – against a waitlist control group, in participants with sleep-maintenance insomnia.
- Ellis et al. (2015): A randomised controlled trial assessing the feasibility and efficacy of a single-session CBT-I intervention versus waitlist control in participants presenting with symptoms of acute insomnia (i.e., insomnia lasting less than 3 months).

*Effect sizes computation*

For each study, between-group effect sizes were computed by comparing each CBT-I treatment format with the corresponding control condition. Given the relatively small sample sizes in the included studies, we calculated unbiased Hedges’ *g* values (Lakens, 2013).

First, change scores were computed for each group as the difference between pre-treatment and post-treatment means (i.e., baseline minus post-treatment). Then, the between-group difference in change scores (Δ) was obtained by subtracting the control group’s change score from that of the treatment group.

To standardize the difference, we calculated the pooled standard deviation (SD) of the post-treatment scores across treatment and control groups as ${SD}_{pooled}= \sqrt{\frac{{SD}_{1}^{2}+ {SD}_{2}^{2}}{2}}$ . As standard deviations of change scores were not available from the original reports, we used the pooled standard deviation of post-treatment scores as a pragmatic approximation, assuming similar pre-post correlations across groups.

The Cohen’s d effect size was then derived by dividing the difference in change scores by this pooled SD, i.e., $d= \frac{\Delta}{{SD}_{pooled}}$ . Finally, we applied a correction for small sample bias to obtain Hedges’ *g*, using the standard adjustment factor based on the pooled degrees of freedom:

$$g=d \times\left( 1 - \frac{3}{4\left( n_{1}+ n_{2} \right) -1} \right)$$

The resulting effect sizes are reported in **Table S1**.

**Table S1.** Between-group effect sizes for single- and multi-session CBT-I interventions on Insomnia Severity Index scores in eligible multiple-arm studies.

| **Study** | **Comparison** | **Cohen’s *d*** | **Hedges’ *g*** |
| --- | --- | --- | --- |
| Amra et al. (2023) | Single-session CBT-I (n = 31) vs. no-treatment control (n = 26) | 1.26 | 1.24 |
| Edinger et al. (2007) | One-session (n = 16) CBT-I vs. waitlist (n = 11) | 1.0 | 0.97 |
| Edinger et al. (2007) | Two-session CBT-I (n = 18) vs. waitlist (n = 11) | 0.32 | 0.31 |
| Edinger et al. (2007) | Four-sessions CBT-I (n = 23) vs. waitlist (n = 11) | 1.76 | 1.72 |
| Edinger et al. (2007) | Eight-session CBT-I (n = 17) vs. waitlist (n = 11) | 1.1 | 1.06 |
| Ellis et al. (2015) | Single-session CBT-I (n = 20) vs. waitlist (n = 20) | 0.51^a^ | 0.5^a^ |
| ^a^: Post-treatment Insomnia Severity Index scores were unavailable, so follow-up assessment scores were considered instead. | | | |
| *Note*. CBT-I: cognitive-behavioural therapy for insomnia. | |  |  |

*Discussion*

Overall, the single-session CBT-I interventions examined across the three included studies yielded moderate to large between-group effect sizes. Specifically, Hedges’ g was 0.50 in Ellis et al. (2015), 0.97 in Edinger et al. (2007), and 1.24 in Amra et al. (2023). While the smaller effect size observed in Ellis et al. may reflect the greater variability and reduced responsiveness associated with acute insomnia, the other studies showed robust effects in favour of single-session interventions. Notably, in Edinger et al. (2007), the effect size for the one-session CBT-I condition exceeded that of the two-session format, was comparable to the full eight-session protocol, and was only outperformed by the four-session format.

Compared to benchmark estimates from meta-analyses (*g* = 0.64 for CBT-I on ISI in the general population at three-month follow-up, and *d* = 0.96 for brief behavioural treatment for insomnia in middle-aged and older adults Kwon et al., 2022; van der Zweerde et al., 2019), the results observed here are encouraging. However, the relatively small sample sizes, heterogeneous definitions of insomnia, and lack of active control conditions across studies represent notable limitations and should be considered when interpreting these findings.

**References**

Amra, B., Ghadiry, F., Vaezi, A., Nematollahy, A., Radfar, N., Haghjoo, S., Penzel, T., & Morin, C. M. (2023). Effect of one-shot cognitive behavioral therapy on insomnia and heart rate variability of health care workers at the time of COVID-19 pandemic: A randomized controlled trial. *Sleep & Breathing = Schlaf & Atmung*, *27*(4), 1411–1418. https://doi.org/10.1007/s11325-022-02746-4

Bastien, C. H., Vallières, A., & Morin, C. M. (2001). Validation of the Insomnia Severity Index as an outcome measure for insomnia research. *Sleep Medicine*, *2*(4), 297–307. https://doi.org/10.1016/s1389-9457(00)00065-4

Edinger, J. D., Wohlgemuth, W. K., Radtke, R. A., Coffman, C. J., & Carney, C. E. (2007). Dose-response effects of cognitive-behavioral insomnia therapy: A randomized clinical trial. *Sleep*, *30*(2), 203–212. https://doi.org/10.1093/sleep/30.2.203

Ellis, J. G., Cushing, T., & Germain, A. (2015). Treating Acute Insomnia: A Randomized Controlled Trial of a “Single-Shot” of Cognitive Behavioral Therapy for Insomnia. *Sleep*, *38*(6), 971–978. https://doi.org/10.5665/sleep.4752

Kwon, M., Wang, J., Wilding, G., Dickerson, S. S., & Dean, G. E. (2022). Brief Behavioral Treatment for Insomnia: A Meta-Analysis. *Behavioral Sleep Medicine*, *20*(6), 674–694. https://doi.org/10.1080/15402002.2021.1982715

Lakens, D. (2013). Calculating and reporting effect sizes to facilitate cumulative science: A practical primer for t-tests and ANOVAs. *Frontiers in Psychology*, *4*. https://doi.org/10.3389/fpsyg.2013.00863

van der Zweerde, T., Bisdounis, L., Kyle, S. D., Lancee, J., & van Straten, A. (2019). Cognitive behavioral therapy for insomnia: A meta-analysis of long-term effects in controlled studies. *Sleep Medicine Reviews*, *48*, 101208. https://doi.org/10.1016/j.smrv.2019.08.002
